# Vulnerability in processing definiteness: The case of heritage Turkish

**DOI:** 10.3389/fpsyg.2023.1286407

**Published:** 2023-12-21

**Authors:** Serkan Uygun

**Affiliations:** Faculty of Educational Sciences, Department of English Language Teaching, Bahçeşehir University, Istanbul, Türkiye

**Keywords:** heritage speakers, Turkish, definiteness, sentence processing, external interface

## Abstract

Definiteness has been argued to be difficult for language learners to acquire because the correct usage of definiteness requires the integration of external interfaces that involve linguistic and non-linguistic information. Previous offline studies with heritage speakers have constantly reported difficulties in the use of pragmatically appropriate definite forms. The current study aims to investigate the processing of definiteness in Turkish heritage speakers via a self-paced reading experiment and to compare heritage speakers' reading times and end-of-sentence acceptance percentage to monolingually-raised Turkish speakers. The results of the experiment indicate significant differences between the heritage and monolingually-raised Turkish speakers only in the end-of-sentence acceptance percentage data but not in the reading time data. This difference between the two groups suggests that Turkish heritage speakers perform differently from the monolingually-raised Turkish speakers only in indefinite-specific sentences when they have to use their metalinguistic knowledge. These results show that heritage speakers experience vulnerability/difficulty when they have to integrate external interfaces in indefinite-specific sentences but not in definite-specific sentences.

## 1 Introduction

The number of people who speak more than one language has been increasing for different reasons, such as population movement, education, and globalism. Jayanath (2021) reports that 43% of people worldwide speak more than one language, and 13% speak more than two languages. These figures clearly outnumber the percentage of monolingual speakers in the world, which is only 40%. These numbers have drawn the attention of researchers, and more research has started to be conducted on bilingual speakers. One of the main questions about bilingualism has been how bilinguals acquire language structures and if they differ from monolingual speakers in their language use and comprehension. Some early research from the 1980s and 1990s found that while lexicon and morphology were more vulnerable to transfer effects, syntactic domains seemed to be less problematic (e.g., Lambert and Freed, 1982; Håkansson, 1995). Sorace and Filiaci (2006) have examined this issue from a generative grammar framework, and they have proposed the Interface Hypothesis (IH).

The original version of the IH predicts that bilinguals can completely acquire narrow syntactic properties, although they may exhibit developmental delays. However, bilinguals will show more vulnerability and therefore may not fully acquire properties involving syntax and other cognitive domains known as interface. A linguistically principled distinction between the interfaces was made in the revised version of the IH (Sorace and Serratrice, 2009). In this version, the term interface was classified as internal and external interfaces based on the type of mapping, whether it is between two linguistic domains or between a linguistic and a non-linguistic domain. Interfaces between linguistic domains such as syntax, semantics, morphology, and phonology and their interactions, such as morphosyntax, are labeled as internal interfaces. On the other hand, external interfaces involve the interaction between linguistic and non-linguistic domains that are related to general cognition and/or world knowledge, such as discourse and pragmatics. The revised version of the IH anticipates only external interfaces (e.g., syntax-pragmatics), in which contextual information is mapped onto the grammar, to be more vulnerable and to pose more difficulties. The reason for the proposed difficulties is that external interfaces not only involve the integration of various types of knowledge across domains, but they also require the simultaneous processing of linguistic and non-linguistic domains, leading to a higher processing load (Laleko and Polinsky, 2013). Sorace and Serratrice (2009) conclude that the challenges posed by internal interfaces differ from those posed by external interfaces. While the former involves features that are internal to the grammar, the latter involves features that are external to the grammar and requires a higher level of language use because it integrates domains outside of the formal grammar (Tsimpli and Sorace, 2006). According to Sorace (2011), bilinguals have less detailed knowledge or less automatic access to computational constraints within the language module, which also makes the external interfaces more vulnerable and difficult for bilinguals. In addition, the simultaneous processing of different domains at external interfaces also puts a strain on the processors of bilinguals, who have fewer cognitive resources available. Sorace (2011) also claims that the IH makes explicit claims for the heritage speakers (HS) at the level of ultimate attainment, which makes HS an important testing ground for the claims of the IH (Montrul and Polinsky, 2011). HS are defined as bilinguals living in a social and familial setting with a different language (i.e., a minority language) from that of the majority of speakers surrounding them (Valdés, 2005). Recent work with HS suggests that while syntax proper is resistant to heritage language conditions, areas where syntax interfaces with other cognitive or non-linguistic domains, such as discourse-pragmatic, are less resilient (Montrul, 2004; Benmamoun et al., 2013; Montrul et al., 2015).

Another crucial question in research on bilingualism has been how bilinguals represent and process linguistic structures that require the integration of knowledge from different linguistic domains. A typical example of this linguistic structure is definiteness (Polinsky, 2018). While the difference between a definite and indefinite noun phrase (NP) is accepted to lie at the syntax-semantics interface (Pérez-Leroux et al., 2004; Espinal, 2010; Borik and Espinal, 2015), external interfaces such as morphosyntax and discourse-pragmatics also play a crucial role in the appropriate use of definiteness when the NP's referent is introduced in the previous context. This is also known as the “uniqueness” (Hawkins, 1984) or “familiarity” (Heim, 1982) requirement. According to Hawkins (1984), a definite entity refers to a unique one, which entails that there is one entity in the world that matches and satisfies the description of the noun. Heim (1982) states that definiteness refers to familiarity, which requires that the entity be known by the speaker and hearer through linguistic introduction or extra-linguistic factors such as contextual salience. According to Aissen (2003), definite NPs are subject to a familiarity requirement, in which the value assigned to the NP referent is determined by previous discourse. Conversely, indefinite NPs are subject to a novelty requirement, but the degree to which the value assigned to the discourse referent can vary. It is more fixed when the value must be chosen from a familiar set, and it is freer when the value can be chosen freely. Therefore, the proper identification of an NP is not only crucial for the hearer to determine what the speaker has in mind but also for the speaker to structure his/her own discourse. This means that the choice of the definite vs. indefinite form of the NP does not merely depend on the morphosyntactic rules but also depends on the discourse-pragmatic cues. Vulnerability/difficulty of morphosyntax in linguistic domains that are regulated by discourse-pragmatic factors in HS have been the subject of research in recent years, and the results suggest special difficulties for its acquisition and target-like use (Laleko, 2010).

Not much is known about the possible challenges of definiteness distinctions in HS. Some studies only focus on the correct usage of definites by investigating the internal interfaces of syntax and semantics. For example, Montrul and Ionin (2010, 2012) investigated the linguistic competence of Spanish HS living in the USA in interpreting Spanish definite articles. The results showed that Spanish HS were treating the articles differently because they interpreted plural definites as specific rather than generic. The authors interpreted these results as a transfer effect from the dominant language because in English, plural definites have a specific reading. There are also several studies that investigate the distinction between definite and indefinite NPs and their pragmatically appropriate use by focusing on the use of external interfaces. In one of these studies, Fenyvesi (2005) explored the use of definiteness marking in Hungarian HS living in the USA and reported a mixing of the two conjugations, that is, the use of definite conjugation in place of indefinite conjugation and vice versa. Finally, Aalberse and Moro (2014) examined the use of definiteness marker in Malay HS living in the Netherlands. They observed an overuse of the definiteness marker and concluded that the Malay HS were under the influence of Dutch, which has an obligatory use of the definite article. The findings of the studies focusing on the external interfaces suggest that HS have more vulnerability/difficulty in the pragmatically appropriate use of definiteness and the integration of the linguistic and non-linguistic domains; in other words, the external interface is challenging and causes difficulties for HS.

### 1.1 Definiteness in Turkish

If an NP is the subject of a default SOV sentence in Turkish, it is used in the nominative case (i.e., zero-marked). Turkish has no obligatory articles that determine the definiteness of the NP in the subject position (Küntay, 2002). However, for NPs in the direct object position, the interpretation of definiteness depends on case marking and indefinite numerals (Taylan and Zimmer, 1994). NPs in the direct object position can be ± definite and ± specific (Coşkun Kunduz and Montrul, 2022). All definite NPs in the direct object position take the accusative marker *-(y)I* and its vowel harmony variant (*-(y)i, -(y)ı, -(y)u*, and *-(y)ü*).^1^ Turkish marks all definite NPs, including proper names, personal pronouns, demonstrative nouns, and definite common nouns (Krause and von Heusinger, 2019), as shown in (1) below, which is taken from Enç (1991, p. 9):

(1) *Zeynep Ali'yi*xx /*on-u*xx /*adam-ı*xx /*o masa-yı*
Zeynep Ali-ACC^2^xx /he-ACCxx /man-ACCxx /that table-ACCxx *gör-dü*.
see-PST.3SG
“Zeynep saw Ali/him/the man /that table.”

The example in (1) shows that Turkish does not have a definite article. However, Turkish has an indefinite article *bir* “a(n)”, which is homophonous to the numeral *bir* “one” but with a different distribution (Kornfilt, 1997). The presence or absence of the accusative marker on indefinite NPs is optional, and it is determined by the specificity of the NP (Enç, 1991; von Heusinger and Kornfilt, 2005), which is determined by the discourse/pragmatics issues (Göksel and Kerslake, 2005). Indefinite NPs are accusative-marked when they are specific and unmarked when they are not specific, and this variation is characterized as the differential object marking phenomenon (Erguvanlı, 1984; Dede, 1986; Enç, 1991; Kornfilt, 1997; von Heusinger and Kornfilt, 2005; Krause and Roberts, 2020). Aissen (2003) states that differential object marking in Turkish distinguishes specifics from non-specifics. The four types of NPs in the direct object position are non-referential or incorporated (2), definite-specific (3), indefinite-non-specific (4), and indefinite-specific (5), which are shown below with examples taken from Coşkun Kunduz and Montrul (2022, p. 605):

(2) Non-referential or incorporated
*Ebru*     *elma*               *ye-di*.
Ebru     apple              eat-PST.3SG
“Ebru was eating an apple/apples (Ebru did apple-eating).”
(3) Definite-specific
*Ebru*     *elma-yı*           *ye-di*.
Ebru     apple-ACC     eat-PST.3SG
“Ebru ate the apple.”
(4) Indefinite-non-specific
*Ebru*      *bir*                   *elma*            *ye-di*.
Ebru       a                     apple           eat-PST.3SG
“Ebru ate an apple.”
(5) Indefinite-specific
*Ebru*     *bir*                   *elma-yı*         *ye-di*.
Ebru     a                     apple-ACC    eat-PST.3SG
“Ebru ate a (certain) apple.”

As can be seen from the examples presented above, when Turkish NPs in the direct object position are accusative-marked, they are specific (examples 3 and 5). However, when they are not accusative-marked, they are non-specific (examples 2 and 4). The example in (2) refers to *apples* in general with a non-referential or incorporated reading because it lacks both the accusative marker and the indefinite article. Conversely, the accusative marked NP in example (3) is definite-specific and indicates that the NP is identifiable to the speaker and the hearer. The indefinite article *bir* in example (4) shows that the NP is indefinite, and the lack of the accusative marker indicates non-specificity, which refers to any member of the category of apples that is not identifiable to the hearer. In example (5), however, the indefinite article *bir* together with the accusative marker implies that the NP is indefinite but specific, which means that the NP is identifiable to the speaker but not to the hearer.

The present study mainly focuses on definite-specific (3) and indefinite-specific (5) conditions. Therefore, it is important to note that both conditions refer to a particular referent (Krause and von Heusinger, 2019) and have a singular meaning because the accusative marker indicates singularity as well (Laszakovits, 2013). However, the main difference between these two conditions is that the hearer can clearly identify the definite-specific NP but not the indefinite-specific NP (Krause and von Heusinger, 2019).

Another important factor in distinguishing between the definite-specific and indefinite-specific conditions is related to the plurality of the NP in the previous discourse. Consider a discourse, where (6) is the first sentence uttered and where the interlocutors have no information other than the common ground established in (6). This sentence can only be followed by (7) because the definite-specific condition is the appropriate use for the second mention of the singular NP *bir çocuk* “a child” introduced in (6). The definite-specific NP ç*ocuğu* “the child” indicates or refers to the *certain* child that was introduced in the previous discourse (Enç, 1991, p. 6).

(6) *Odam-a*               *bir*                         *çocuk*
my room-DAT    a                            child
*gir-di*.
enter-PST.3SG
“A child entered my room.”
(7) *Çocuğ-u*              *hemen*                   *tanı-dı-m*.
child-ACC          immediately         recognize-PST-1SG
“I immediately recognized the child.”

However, in discourse (8), the NP is introduced in its plural form; therefore, this sentence can only be followed by (9) since the second mention of the plural NP ç*ocuklar* “children” can be made via the use of indefinite-specific condition. According to Enç (1991, p. 6), the indefinite-specific NP *bir çocuğu* “one of the children” is appropriate because it is about the child, who is included in the set of children established by the previous discourse.

(8) *Odam-a*                   *çocuklar*                 *gir-di*.
my room-DAT        children                 enter-PST.3SG
“Children entered my room.”
(9) *Bir*                           *çocuğ-u*                  *hemen*
a                              child-ACC              immediately
*tanı-dı-m*.
recognize-PST-1SG
“I immediately recognized one of the children.”

The same discourse (8) can also be followed by (10), which includes a partitive NP and is equivalent to (9) in terms of meaning (Enç, 1991, p. 6).

(10) *Çocuk-lar-dan*               *bir-i-ni*                     *hemen*
child-PL-ABL                one-POSS-ACC       immediately
*tanı-dı-m*.
recognize-PST-1SG
“I immediately recognized one of the children.”

### 1.2 Previous studies in Turkish

Previous studies reveal that Turkish-speaking children acquire the accusative marker quite early, at the age of 2;0 and use it with very few errors in their speech production (Slobin and Bever, 1982; Aksu-Koç and Slobin, 1985; Ketrez, 1999; Ketrez and Aksu-Koç, 2009; Özge et al., 2019). However, its early use in children is restricted to definite objects, although it can be used with a variety of different interpretations in adult speech. These interpretations involve discourse-linking (Nilsson, 1985; Enç, 1991; Zidani-Eroğlu, 1997), specificity (von Heusinger, 2002; von Heusinger and Kornfilt, 2005), presuppositionality (Kennelly, 1997; Kelepir, 2001), individuation/particularization (Nilsson, 1985; Taylan and Zimmer, 1994; Bolgün, 2005; Kılıçaslan, 2006), and totality/delimitedness (Nilsson, 1985; Nakipoğlu, 2009). In addition, the indefinite article *bir* is acquired much later than the accusative marker, at around 7;0 years of age (Küntay, 2002; Ketrez, 2015). For example, Küntay (2002) investigated how indefinite referents were expressed in preschool children, elementary school children, and adults on a picture series task. The task was a wordless picture story called “Balloon Story”, which consisted of six frames that were presented as two three-picture strips. In the story, a little boy encounters a balloonman while walking and buys a balloon. Later, the balloon flies off, and the boy starts crying. The child participants were first asked to go through the pictures quietly to become familiar with the plot. They were also told that they would tell the story to someone else who was not in the room at that moment. After completing this phase, the listener was invited to the room and was seated on the opposite side of the table from the child. After that, the child was instructed to tell the listener what had happened in the story from the beginning. The results of this elicitation task revealed that preschool children used the indefinite article *bir* much less frequently than the older speakers, indicating that Turkish-speaking children do not acquire the correct usage of *bir* until around 7 years of age. In another study, Ketrez (2015) tested 31 children between the ages of 3;5 and 6;6 together with 25 adults in a comprehension experiment to measure their knowledge of accusative-marked indefinite objects. The results suggested that even at age 6, children were not able to interpret the accusative-marked indefinites like adult Turkish speakers (80 vs. 99%). The researcher interpreted these results as incomplete acquisition of the accusative case and suggested two possible reasons for this failure: one of them is the complexity of differential object marking in Turkish, and the other is the infrequent use of indefinite-specific structures and relevant contexts in child-directed speech. These acquisition studies reveal that accusative-marked indefinites are one of those structures that are acquired at later stages because of the complexity of the structure and its infrequent use in child-directed speech. Therefore, Ketrez (2015) suggests that the acquisition of this complex structure requires not only more time but also a different type of input.

Research on definiteness has also been conducted with Turkish HS. While some of these studies focus on the differential object marking phenomenon (Krause and von Heusinger, 2019; Krause and Roberts, 2020; Coşkun Kunduz and Montrul, 2022), there are also studies that mainly explore the knowledge and use of the indefinite marker *bir* in Turkish (Backus et al., 2011; Felser and Arslan, 2019; Yılmaz and Sauermann, 2023). For example, Backus et al. (2011) rest their discussion on the findings of Doğruöz (2007) and Doğruöz and Backus (2007, 2009), which are based on a corpus of spoken heritage Turkish collected in the Netherlands. They found that Turkish HS had difficulties with the correct usage of definite-specific vs. indefinite-specific NPs. In example (11), the heritage participant uses the phrase *akustik bir gitar* “an acoustic guitar”, when trying to refer to his friend's specific guitar, while a monolingually-raised speaker of Turkish would use the phrase *akustik gitar* without the indefinite marker *bir* as in example (12) to convey this meaning. The authors attribute this to the effect of Dutch, which uses an indefinite article in the translation of this sentence. A monolingually-raised Turkish speaker would interpret example (11) as contrasting acoustic with electric guitars (Backus et al., 2011, p. 742).

(11) Heritage                           Turkish
*Akustik*                              *bir*                     *gitar*               *var*       *on-da*.
acoustic                             a                       guitar              exist     he-LOC
“He has an acoustic guitar.”
(12) Monolingually-raised      Turkish
*Akustik*                               *gitar*                 *var*                 *on-da*.
acoustic                              guitar               exist               he-LOC
“He has an acoustic guitar.”

In another study, Felser and Arslan (2019) used an untimed multiple-choice discourse-completion task to investigate if Turkish HS could select the appropriate definite and indefinite forms in different discourse contexts. They found that HS living in Germany had difficulties in providing appropriate responses for each definiteness condition when compared to monolingually-raised Turkish speakers (MS); that is, more indefinite responses in the definite condition and more definite responses in the indefinite condition. Finally, Yılmaz and Sauermann (2023) compared Turkish HS living in Germany to a MS group in Turkey via an untimed elicitation task where the participants had to choose the correct form of the NP in the direct object position after reading a dialogue. The participants were presented with three forms of the NP: accusative-marked indefinite NP, unmarked indefinite NP, and accusative-marked definite NP. The results revealed no difference between the two groups. Turkish HS were able to successfully encode/decode relationships, construct pragmatically appropriate utterances, and make similar preferences to those of MS.

To recapitulate, previous studies with Turkish HS are scarce, and this phenomenon has been investigated via offline methods such as elicitation tasks, informal interviews, and multiple-choice completion tasks. These studies inform us about the metalinguistic judgments of the HS when there is no time limitation. The results are inconclusive and cannot provide further evidence of the vulnerability/difficulty in integrating external interfaces observed in HS in general.

### 1.3 The present study

While previous research with Turkish HS has employed offline methods to examine the choice of definite vs. indefinite NPs, the current study aims to investigate if external interfaces such as morphosyntax and discourse/pragmatics are vulnerable/difficult for HS during real-time sentence processing by exploring the reading times (RTs) of the NPs and their end-of-sentence acceptance percentages in a self-paced reading experiment. Since offline tasks do not provide direct access to one's mental processes as they unfold in real time, an online task was used because online tasks capture the automatic responses of the participants and enable the researchers to get more direct access to *how* language processing unfolds in real time (Bayram et al., 2021). A self-paced reading experiment asks its participants to read sentences on the screen one word/phrase at a time and to press a button to move on to the next word/phrase. The main assumption is that the total amount of time to read a word or phrase reflects the total amount of time to process that word or phrase, and longer RTs mean processing difficulty (Jegerski, 2014). According to Bayram et al. (2021), the main goal of a self-paced experiment is not to compare the RTs of the HS and MS on a quantitative basis but to explore and understand if the HS process their heritage language qualitatively differently from the MS group, which serves as the control group. Rothman et al. (2023) recently suggested that the inclusion of monolingual control groups for studying heritage language bilingualism has had detrimental effects on understanding the grammar of HS holistically. While HS usually receive no formal training in the standard version of their heritage language, the participants in the monolingual control group receive substantial training in it, which alone adds noise and makes the comparison uncontrolled. However, a monolingual control group is necessary in the current study to understand how definiteness and plurality interact with each other in this group so that comparisons can be made with the HS. With the inclusion of a monolingual control group, it becomes possible to explore if the interaction of definiteness and plurality of the NP in the context sentence affects HS in the same way or differently.

The current study addresses the following research questions:

1. How do Turkish HS process the definite and indefinite NPs in singular/plural contexts? Do their reading times differ from the MS group?
2. What is the end-of-sentence acceptance percentage for the HS group? Does their acceptance percentage differ from the MS group?

The aim of the first research question is to see how the RTs of the NPs are affected by the morphosyntactic and discourse/pragmatic information and to compare the groups' implicit processing routes. The motivation for the second research question is to explore and compare the groups' metalinguistic knowledge. If differences are observed between HS and MS groups in their RTs and end-of-sentence acceptance percentages, this would provide support for the revised version of the IH (Sorace and Serratrice, 2009), indicating that external interfaces are vulnerable/difficult for HS and pose difficulties to acquire and process. If there are no differences between the HS and MS groups in their RTs and end-of-sentence acceptance percentages, this would refute the predictions of IH and suggest that HS can use the discourse-pragmatic cues correctly and have no difficulties in integrating linguistic and non-linguistic domains. If differences are only observed in RTs but not in end-of-sentence acceptance percentages, this would suggest that HS face difficulties with external interfaces only in implicit processing but not in using their metalinguistic knowledge. Finally, if there are differences between HS and MS groups only in their end-of-sentence acceptance percentages but not in their RTs, this would indicate that HS experience difficulties in integrating linguistic and non-linguistic information only when they have to use their metalinguistic knowledge to make a judgment about the sentences they read.

## 2 Materials and methods

### 2.1 Participants

The MS group consisted of 40 participants who were born and raised in Turkey and had never lived abroad. All MS participants were recruited and tested in Istanbul, Turkey. They were either university graduates or studying at the university at the time of testing, and they all spoke the standard dialect of Turkish. One MS participant had to be excluded due to high error rates (>30%) in the filler condition. The data of the remaining 39 MS participants (mean age = 36.87, SD = 9.21, age range = 19–60, 29 females) were analyzed. The Turkish HS group involved 60 participants who were exposed to Turkish from birth. They spoke Turkish and German in their daily lives and were recruited from the large Turkish communities in Berlin and Potsdam. One participant from the HS group was excluded due to low Turkish proficiency (below 12 out of 20), which indicates a proficiency level lower than B2 level based on the Common European Framework of Reference (CEFR). B2 level was used as a cut-off point because learners at this level are considered “independent” learners, who are able to understand and be understood in most situations. The Turkish TELC (The European Language Certificates) test was applied to the HS group, and the language structure part of the test consists of two cloze tests with 20 questions in total. As a result, the data of 59 HS participants (mean age = 27.78, SD = 6.06, age range = 19–50, 42 females) were put into analysis. All HS participants completed a background questionnaire, which was adapted from the Language Experience and Proficiency Questionnaire (LEAP-Q), originally developed by Marian et al. (2007). The mean age of acquisition of German in the HS group was 3.01 (SD = 1.85, age range = 0–6), which indicates that all HS were exposed to German before starting school. The HS group also had a high score from the Turkish TELC test (mean score = 18.44, SD = 1.62, score range = 13–20), and the results of their self-ratings showed a predominant use of Turkish in a normal week in terms of percentage (mean percentage = 61.61%, SD = 21.82, range = 15–90%). In addition, HS were asked to self-rate their proficiency in their Turkish language skills, and the results indicate a high proficiency level out of 10 (Speaking: mean = 7.91, SD = 1.63; Listening: mean = 8.84, SD = 1.17; Writing: mean = 7.17, SD = 2.04; Reading: mean = 8.21, SD = 1.70). The self-rating scores, together with the TELC scores, reveal that the HS group had a high level of proficiency in Turkish. The HS group also self-rated their proficiency in German language skills, and the results exhibit a high proficiency level in German as well (Speaking: mean = 9.28, SD = 0.91; Listening: mean = 9.62, SD = 0.64; Writing: mean = 9.24, SD = 1.10; Reading: mean = 9.62, SD = 0.79). All participants received a small fee for their participation.

### 2.2 Materials

By manipulating definiteness (definite-specific vs. indefinite-specific) and plurality (plural vs. singular NP), 24 experimental sentence sets in four different conditions were created, as illustrated in (13–16). All experimental sentences followed a context sentence, which had two different versions: one with a plural NP and one with a singular NP. In all context sentences, the NP was always inanimate. The continuation sentence contained either a definite-specific or an indefinite-specific NP, which needed to be determined by the discourse-pragmatic cue presented in the context sentence. The context sentence below for examples (13) and (14) presents a plural NP, *kitaplar* “books”. Because the NP was previously presented in its plural form, it is more appropriate to use the NP in its indefinite-specific form (*bir kitabı* “one of the books”) as in example (14), while it is inappropriate to use the definite-specific form presented in example (13). In Enç's (1991) account, example (14) is more appropriate in this context because the indefinite-specific NP (*bir kitabı* “one of the books”) refers to the presence of a superset of discourse referents introduced in the context sentence (*kitaplar* “books”).

Context sentence for Plural NPs:
*Masa-nın*                   *üzer-in-de*                 *kalın*               *kitap-lar*
table-GEN                 on-POSS-LOC         thick               book-PL
*var-dı*.
to be-PST.3SG
“There were thick books on the table.”
(13) Definite-specific – Plural (DS-PL):
*Can-ı*                         *çok*                           *sıkıl-an*            *Ayşe*
Ø-ACC                     very                          bored-REL     Ayşe
*kitab-ı*                       *oku-du*.
book-ACC                read-PST.3SG
“Ayşe, who was very bored, read the book.”
(14) Indefinite-specific – Plural (IS-PL):
*Can-ı*                        *çok*                             *sıkıl-an*           *Ayşe*    *bir*
Ø-ACC                    very                            bored-REL    Ayşe     one
*kitab-ı*                       *oku-du*.
book-ACC                read-PST.3SG
“Ayşe, who was very bored, read one of the books.”

The context sentence for examples (15) and (16) presents the NP in its singular form, *bir kitap* “a book”. As a result of the prior mention of this singular NP, it is more appropriate to use this NP in its definite-specific form (*kitabı* “the book”), as in example (15), because according to Enç (1991), its referent is directly linked to a previously related discourse referent (*bir kitap* “a book”). Yet, the indefinite-specific form presented in example (16) would be inappropriate to use.

Context sentence for Singular NPs:
*Masa-nın*                    *üzer-in-de*                    *kalın*                    *bir*
table-GEN                  on-POSS-LOC            thick                    a
*kitap*                           *var-dı*.
book                           to be-PST.3SG
“There was a thick book on the table.”
(15) Definite-specific – Singular (DS-SG):
*Can-ı*                         *çok*                               *sıkıl-an*                *Ayşe*
Ø-ACC                      very                             bored-REL         Ayşe
*kitab-ı*                        *oku-du*.
book-ACC                 read-PST.3SG
“Ayşe, who was very bored, read the book.”
(16) Indefinite-specific – Singular (IS-SG):
*Can-ı*                          *çok*                             *sıkıl-an*                  *Ayşe*      *bir*
Ø-ACC                      very                            bored-REL            Ayşe      one
*kitab-ı*                        *oku-du*.
book-ACC                 read-PST.3SG
“Ayşe, who was very bored, read one of the books.”

Four different presentation lists were created in a Latin-square design, and the items in each version were pseudo-randomized. The experimental items were mixed with 48 filler sentences, and a total of 72 items were used for each list. All of the filler sentences had the same form as the experimental sentences; that is, they also had a context sentence and a continuation sentence that measured subject-verb agreement marking and tense marking. Half of the filler sentences were correct.

### 2.3 Design and procedure

The experiment was prepared and run on Ibex Farm (Drummond, 2013), which is a web-based platform for hosting psycholinguistic experiments. By using the non-cumulative moving window paradigm (Just et al., 1982), the sentences in the experiment were presented word-by-word. In the beginning of each trial, all words in the sentence were masked by underscores. When the participant pressed the space bar button, the first word of the sentence was revealed. By pressing the space bar button again, the first word was masked by an underscore, and the second word was revealed. When participants reached the last word of the sentence, which was followed by a full stop, they had to press the space bar button again and saw the following question: “Is the second sentence a grammatically and semantically good continuation of the context sentence?” Participants had to press the “f” button if their response was “yes” or the “j” button if their response was “no”. After responding to this question, they had to press the space bar button again to see the next trial.

The first part of the experiment involved the demographic background questionnaire and the consent form. Then, the participants had to read the instructions carefully, which were followed by five practice items so that they could familiarize themselves with the procedure. This was followed by the main experiment, in which the participants had to read the sentences carefully and answer the questions as quickly as possible. Participants were sent a link to the experiment, and they completed the test on their personal computers. Web-based testing has recently been preferred by many researchers because it has allowed researchers to reach more participants and provided many reliable results (Gibson et al., 2011; Sprouse, 2011; Enochson and Culbertson, 2015; Chemla et al., 2016; Lago et al., 2019). Participants could keep track of their progress via a progress bar that was placed above the sentences. It took approximately 20 min to complete the experiment, and HS were asked to do the Turkish proficiency test after the experiment.

## 3 Results

Statistical analyses of the participants RT data and end-of-sentence acceptance percentage data were conducted with R, which is an open-source programming language and environment for statistical computing (R Core Team, 2021). Regarding the RT data, the dependent measures were word-by-word RTs, and the main focus was on obtaining significant group differences and/or interactions involving the factor, Group. Data cleaning procedures were applied to the RT data; namely, RTs exceeding 2.5 standard deviations above and below a participant's mean log RT were deemed outliers and removed (HS group = 2.91%; MS group = 3.35%). In order to overcome the instability of the word length and problems related to individual differences in reading times, residual reading times (RRTs) were calculated on the remaining data with linear modeling on the log-transformed RTs. While positive RRTs values refer to slower reading times than expected, negative RRTs values mean faster reading times. The RRTs data were analyzed for two regions of interest: the “Critical Region”, where the NP is in its accusative form, and the “Spillover” region, which is immediately after the Critical Region (see Table 1 for the regions; analyses were conducted for Regions 6 and 7).

**Table 1:** Regions of interest in the experimental sentences.

**Regions**	**Definite-specific**	**Indefinite-specific**	**Example**
Region 1	Relative clause 1	Relative clause 1	Canı (*no translation*)
Region 2	Relative clause 2	Relative clause 2	çok (*very*)
Region 3	Relative clause 3	Relative clause 3	sıkılan (*bored*)
Region 4	Subject	Subject	Ayşe (*Ayşe*)
Region 5	*Not applicable*	Before critical region	bir (*one of*)
Region 6	Critical region	Critical region	kitabı (*the book(s)*)
Region 7	Spillover	Spillover	okudu (*read*)

Linear mixed-effects regression models with crossed random effects for items and subjects were used to analyze the RRTs data (Baayen et al., 2008). The models included the subject-level variable “Group” (HS vs. MS) and item-level variables “Definiteness” (definite vs. indefinite) and “Plurality” (plural NP vs. singular NP) as fixed effects together with random slopes for subject and item. The models were fitted using the package *lme4* (Bates et al., 2015). While sum-coded contrasts (−0.5, 0.5) were employed for the factors Group, Definiteness, and Plurality for the main effects and overall interactions, treatment contrasts were employed for single comparisons. As backwards elimination was employed, a model with maximum random effects and interactions was constructed as a starting point. When the model did not converge, it was gradually simplified until convergence was reached (Barr et al., 2013). In the simplification process, random slopes by subject and item for each fixed effect in the model were only retained if they improved the model fit significantly, which was measured by using the Akaike Information Criterion (AIC). According to Venables and Ripley (2002), AIC provides a measure that penalizes complexity and leads to predictors being kept only when they substantially contribute to explaining variance in the data. Each time, the model with the lower AIC was selected until the simplification process did not produce a model with a lower AIC. The final version of the model included random slopes for plurality by item and by subject. The effect sizes are reported by using model coefficients in log odds (ß), standard errors (*SE*), *t*-statistics, and *p*-values. *P*-values were computed by using the *lmerTest* package and the Satterthwaite's approximation for denominator degrees of freedom (Kuznetsova et al., 2014).

**Figure 1:**
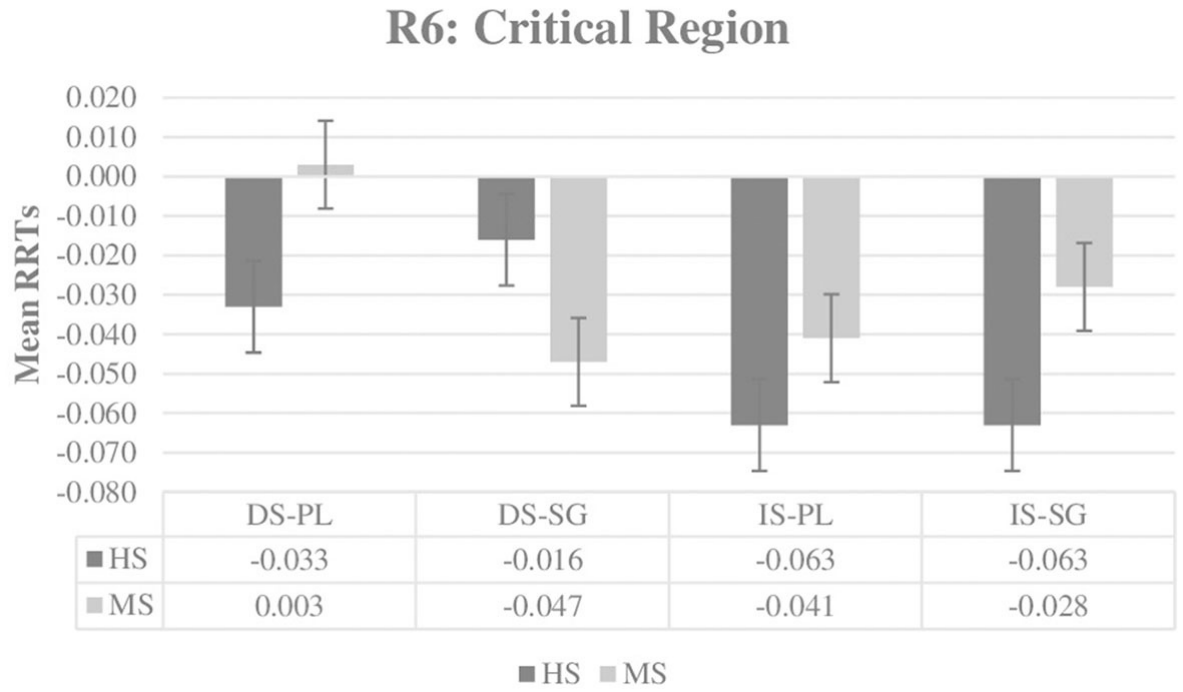
Mean RRTs of both groups for Region 6 (Critical Region). RRTs, Residual reading times; HS, Heritage speakers; MS, Monolingually-raised speakers; DS, Definite-specific; IS, Indefinite-specific; PL, Plural NP; SG, Singular NP.

The first analysis was conducted in Region 6 (Critical Region), where the NP is in its accusative form. Figure 1 provides an overview of the groups' mean RRTs, and Table 2 presents the results of the best-fit model in the Critical Region. The results of the RRTs analysis indicate a significant effect of definiteness only (ß: −0.025, *SE:* 0.010, *t* = −2.411, *p* = 0.016), which reveals that definite-specific NPs receive significantly shorter RTs than indefinite-specific NPs.

**Table 2:** Linear mixed effects model output for Region 6 (Critical Region).

	**ß**	***SE***	***t***	***p***
Intercept	−0.036	0.011	−3.307	**0.000** ^*^
Definiteness (Definite vs. Indefinite)	−0.025	0.010	−2.411	**0.016** ^*^
Plurality (Plural vs. Singular)	0.004	0.012	0.373	0.710
Group (HS vs. MS)	−0.016	0.017	−0.936	0.350
Definiteness^*^Plurality	0.021	0.021	1.040	0.298
Definiteness^*^Group	−0.027	0.021	−1.314	0.188
Plurality^*^Group	−0.027	0.022	−1.207	0.228
Definiteness^*^ Plurality^*^ Group	−0.078	0.041	−1.906	0.056

Formula in R: RTresidual ~ Definiteness ^*^ Plurality ^*^ Group + (1 + Plurality | item) + (1 + Plurality | subject). Bold values indicate significant results. The symbol “^*^” indicates significant results.

**Figure 2:**
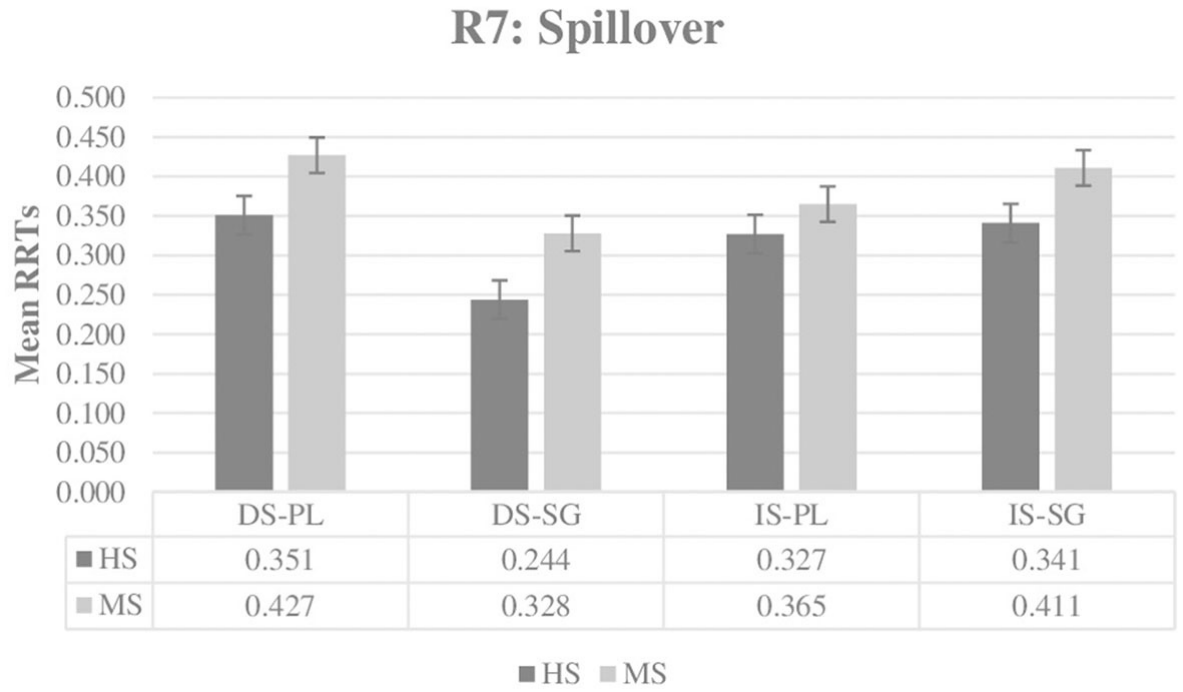
Mean RRTs of both groups for Region 7 (Spillover). RRTs, Residual reading times; HS, Heritage speakers; MS, Monolingually-raised speakers; DS, Definite-specific; IS, Indefinite-specific; PL, Plural NP; SG, Singular NP.

The next and final RRTs analysis was conducted in Region 7 (Spillover), which is immediately after the Critical Region and is also the end of the sentence. Figure 2 illustrates the mean RRTs of both groups, and Table 3 shows the results of the best-fit model in the Spillover Region. In this region, a significant main effect of plurality (ß: 0.047, *SE:* 0.017, *t* = 2.813, *p* = 0.004) and a significant two-way interaction of definiteness and plurality (ß: 0.146, *SE:* 0.029, *t* = 5.101, *p* < 0.001) have been obtained. The main effect of plurality indicates that sentences in plural NP contexts take longer to respond than sentences in singular NP contexts. As can be seen in Table 3A, the significant definiteness and plurality interaction reveals that definite-specific sentences take significantly longer to respond (ß: 0.044, *SE:* 0.020, *t* = 2.252, *p* = 0.024) when the model is releveled for plural NP contexts; however, when the model is releveled for singular NP contexts, indefinite-specific sentences take significantly longer to respond (ß: −0.099, *SE:* 0.020, *t* = −5.066, *p* < 0.001). No significant group differences or interactions involving the factor “Group” were obtained in this region.

**Table 3:** Linear mixed effects model output for Region 7 (Spillover).

	**ß**	***SE***	***t***	***p***
Intercept	−0.036	0.026	−13.595	**0.000** ^*^
Definiteness (Definite vs. Indefinite)	−0.025	0.014	−1.716	0.086
Plurality (Plural vs. Singular)	0.047	0.017	2.813	**0.004** ^*^
Group (HS vs. MS)	−0.077	0.049	−1.578	0.114
Definiteness^*^Plurality	0.146	0.027	5.101	**0.000** ^*^
Definiteness^*^Group	−0.025	0.029	−0.889	0.374
Plurality^*^Group	0.001	0.029	0.009	0.994
Definiteness^*^ Plurality^*^Group	−0.018	0.057	−0.307	0.758

Formula in R: RTresidual ~ Definiteness ^*^ Plurality ^*^ Group + (1 + Plurality | item) + (1 + Plurality | subject). Bold values indicate significant results. The symbol “^*^” indicates significant results.

**Table 3A:** *Post-hoc* analysis for the Definiteness^*^Plurality in Region 7 (Spillover).

	**ß**	***SE***	***t***	***p***
Relevelled for plural NP contexts	0.044	0.020	2.252	**0.024** ^*^
Relevelled for singular NP contexts	−0.099	0.020	−5.066	**0.000** ^*^

Formula in R: RTresidual ~ Definiteness ^*^ Plurality + (1 + Plurality | item) + (1 + Plurality | subject). Bold values indicate significant results. The symbol “^*^” indicates significant results.

Regarding the end-of-sentence acceptance percentage data, responses were coded with two possible options: accept (1) vs. reject (0). A generalized linear mixed-effects regression model (binomial family, with the bobyqa optimizer) was fitted to the participants' verb responses by using the same fixed and random effects. The final version of the model included by item random slope for plurality and by subject random slope for definiteness and plurality interaction. The effect sizes are reported by using model coefficients in log odds (ß), standard errors (*SE*), *z*-statistics, and *p*-values.

**Figure 3:**
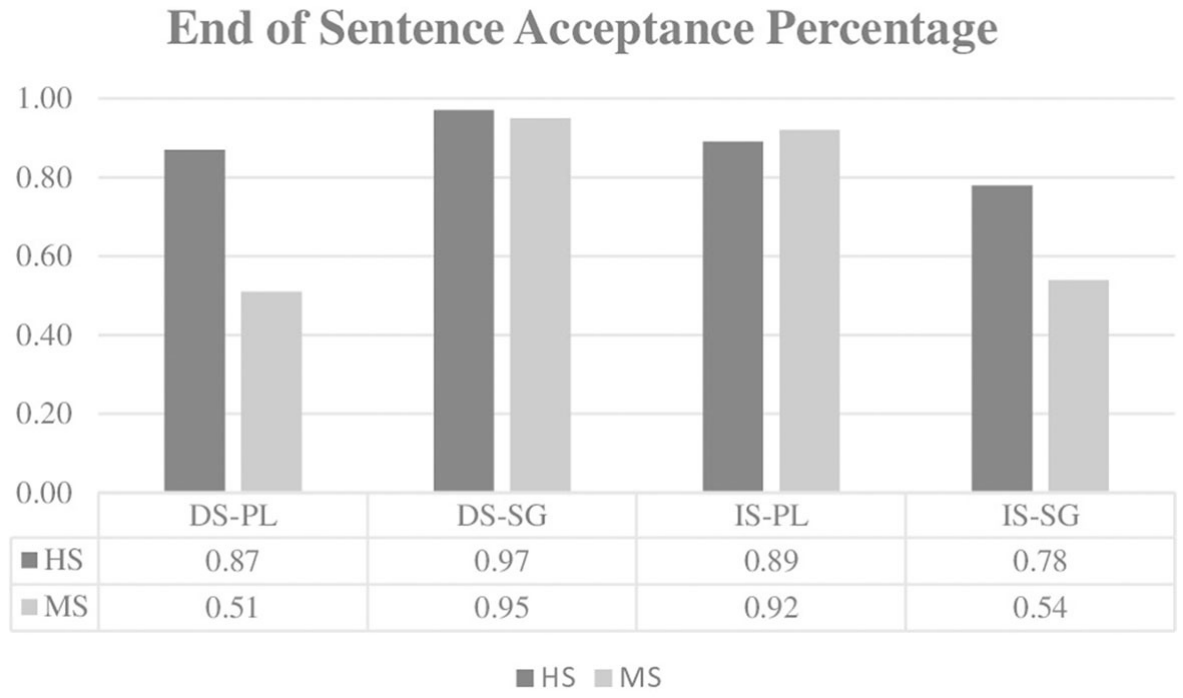
Acceptance percentage of both group for end of sentence response. HS, Heritage speakers; MS, Monolingually-raised speakers; DS, Definite-specific; IS, Indefinite-specific; PL, Plural NP; SG, Singular NP.

Figure 3 displays both groups' acceptance percentages, and Table 4 displays the results of the best-fit model. The end-of-sentence acceptance percentage data analysis reveals a significant main effect of group (ß: 1.400, *SE:* 0.338, *z* = 4.146, *p* < 0.001), significant two-way interactions of definiteness and plurality (ß: −3.789, *SE:* 0.785, *z* = −4.829, *p* < 0.001) and definiteness and group (ß: 0.985, *SE:* 0.450, *z* = 2.011, *p* = 0.044) and a significant three-way interaction of definiteness, plurality, and group (ß: 4.943, *SE:* 1.199, *z* = 4.122, *p* < 0.001). The main effect of group indicates that, in general, the HS group gives a significantly higher percentage of acceptance than the MS group (88 vs. 73%). The interaction of definiteness and plurality shows that in singular NP contexts, definite-specific sentences are accepted significantly more than indefinite-specific sentences in both groups (ß: 2.004, *SE:* 0.373, *z* = 5.379, *p* < 0.001), while this difference is not significant in plural NP contexts. The definiteness and group interaction indicates that the HS group has a significantly higher acceptance percentage than the MS group both in definite-specific (92 vs. 73%; ß: 1.893, *SE:* 0.427, *z* = 4.434, *p* < 0.001) and indefinite-specific (84 vs. 73%; ß: 0.907, *SE:* 0.407, *z* = 2.229, *p* = 0.026) sentences. Finally, the three-way interaction of definiteness, plurality, and group reveals a significant interaction of definiteness and plurality (ß: −6.375, *SE:* 1.125, *z* = −5.665, *p* < 0.001) for the MS group. This interaction for the MS group demonstrates that in plural NP contexts, indefinite-specific sentences are accepted significantly more (ß: −3.285, *SE:* 0.836, *z* = −3.928, *p* < 0.001); however, in singular NP contexts, definite-specific sentences are accepted significantly more (ß: 3.089, *SE:* 0.623, *z* = 4.964, *p* < 0.001). For the HS group, this interaction was not significant (ß: −1.835, *SE:* 1.230, *z* = −1.493, *p* = 0.136).

**Table 4:** Linear mixed effects model output for end of sentence response.

	**ß**	***SE***	***z***	***p***
Intercept	2.443	0.235	10.395	**0.000** ^*^
Definiteness (Definite vs. Indefinite)	0.581	0.358	1.624	0.104
Plurality (Plural vs. Singular)	−0.173	0.364	−0.476	0.634
Group (HS vs. MS)	1.400	0.338	4.146	**0.000** ^*^
Definiteness^*^Plurality	−3.789	0.785	−4.829	**0.000** ^*^
Definiteness^*^Group	0.985	0.450	2.011	**0.044** ^*^
Plurality^*^Group	0.219	0.474	0.462	0.644
Definiteness^*^ Plurality^*^Group	4.943	1.199	4.122	**0.000** ^*^

Formula in R: Answer ~ Definiteness ^*^ Plurality ^*^ Group + (1 + Plurality | item) + (1 + Definiteness ^*^ Plurality | subject). Bold values indicate significant results. The symbol “^*^” indicates significant results.

## 4 Discussion

The present study explored the residual reading times of the noun phrases and the end-of-sentence acceptance percentages in an attempt to investigate if external interfaces such as morphosyntax and discourse/pragmatics are vulnerable/difficult for heritage speakers during real-time sentence processing in comparison to monolingually-raised speakers of Turkish.

When the results of the RRTs data in the Critical Region are considered, there is clear evidence that the HS group patterns with the MS group. Both groups show sensitivity toward the definiteness distinction in this region because sentences with indefinite-specific NPs receive significantly longer RRTs when compared to sentences with definite-specific NPs. The RRTs data in the Spillover Region, which immediately follows the Critical Region, also reveals no difference between the HS and MS groups. In the Spillover Region, the plurality of the NP in the context sentence affects both groups in the same way, with significantly longer RRTs for sentences with plural NP contexts in comparison to singular NP contexts. In addition, the significant definiteness and plurality interaction obtained in the Spillover Region indicates that both groups are influenced by this manipulation in the same way. That is, sentences with plural NP contexts receive significantly slower RRTs for the pragmatically inappropriate definite-specific continuation, and sentences with singular NP contexts receive significantly slower RRTs for the pragmatically inappropriate indefinite-specific continuation.

However, the end-of-sentence acceptance percentage data suggests that HS experience some difficulties here and display several differences when compared to the MS group. First of all, the HS group accepted the sentences significantly more than the MS group (88 vs. 73%), and this significant difference exists both in the acceptance of definite-specifics (92 vs. 73%) and indefinite-specifics (84 vs. 73%). Despite this difference, the significant definiteness and plurality interaction shows that both the HS and MS groups have significantly higher end-of-sentence acceptance percentages with definite-specific sentences in singular NP contexts. Crucially, the significant definiteness, plurality, and group interaction indicates that the MS group also has a significantly higher end-of-sentence acceptance percentage with indefinite-specific sentences in plural NP contexts. Yet, the HS group does not display this sensitivity in plural NP contexts. These results clearly show that while HS partially pattern with MS in their end-of-sentence acceptance percentages, the only significant difference is observed in plural NP contexts, which require pragmatically appropriate indefinite-specific sentence continuation. In plural NP contexts, the HS group also displays a very high end-of-sentence acceptance percentage for the pragmatically inappropriate definite-specific sentence continuation.

How can these results be accounted for? It is important to note that this is a study that aims to investigate definiteness in HS via an online tool that measures the RTs of the participants. The online nature of the task provides additional information about the temporal resolution of processing rather than the metalinguistic knowledge provided by offline tasks (Bayram et al., 2021). And the obtained group differences are expected to provide more insights into the vulnerability/difficulty that HS may experience in real-time sentence processing, namely in integrating knowledge from different linguistic domains. As stated previously, the revised version of the IH makes a clear distinction between two types of interfaces: the internal interfaces have interactions between linguistic domains (e.g., syntax and morphology), whereas the external interfaces involve interactions between linguistic and non-linguistic domains (e.g., syntax and discourse/pragmatics). According to Sorace (2011, 2012), processing limitations are expected to occur in external interfaces because structures that require external mappings are more taxing than structures that require internal mappings. And the online nature of the experimental task can clearly demonstrate at which point it becomes difficult to integrate information from different domains, which might result in different processing patterns (Sorace, 2011). However, the RRTs results in the Critical and Spillover Regions do not provide support for these claims because the HS group does not differ from the MS group in processing definiteness and its interaction with plurality.

On the other hand, in the end-of-sentence questions that follow each experimental item, differences between both groups are observed only in plural NP contexts, which require the pragmatically appropriate indefinite-specific sentence continuation. Why do HS differ significantly from the MS group only in plural NP contexts that need to be followed by indefinite-specific sentences? Recall that Turkish-speaking children acquire the accusative marker at the age of 2;0 with restricted usage for definite objects only. Ketrez (2015) reports that in Frog Story narrations (Turkish-Aksu corpora, at CHILDES database), *bir* is used in indefinite structures without the accusative case in most of its occurrences (e.g., *bir delik* “a/one hole”). In addition, in only one child's narration, *bir* appears with an accusative-marked object at age 5;2 with the intended meaning of a “particular” object (*bir kavanozu* “one of the jars”). Based on these results, Ketrez (2015) concludes that accusative-marked indefinites are acquired at later stages because of the complexity and infrequent use in child-directed speech. According to Putnam and Sánchez's (2013) 4-stage model, late-acquired features in heritage languages may be very weakly activated, which generates a decline in their availability and results in producing structures that are different from monolingual control speakers. The model mainly considers *feature activation* as the main reason why HS differ from MS by claiming that if a feature is sufficiently activated, fewer differences will be observed between HS and MS. In addition, the model argues that if the elements of grammar are less salient and have low frequency in the input, these elements become a recessive feature in the heritage speaker's grammar, leading to restructuring and simplification of the heritage grammar. The findings that Turkish indefinite-specifics are late acquired and have a low frequency in child-directed speech might explain why HS in the present study differ from the MS group only in the indefinite-specific sentences in plural NP contexts.

When the partial differences observed in the end-of-sentence acceptance percentage data were taken into consideration, the results showed the vulnerability/difficulty that HS faced in integrating external interfaces only for indefinite-specific sentences in plural NP contexts. When morphosyntactic information needs to be integrated with discourse/pragmatic information to make a judgment about the sentences being read/processed, HS experience difficulties in making pragmatically appropriate choices only in plural NP contexts, indicating the challenges of the definiteness distinction in this specific condition. This result is partially in line with the previous offline studies comparing HS and MS groups not only in different languages (Fenyvesi, 2005; Aalberse and Moro, 2014) but also in Turkish as a heritage language (Backus et al., 2011; Felser and Arslan, 2019) because these studies report vulnerability/difficulty for both definite and indefinite conditions. Yet, the present result differs from Yılmaz and Sauermann (2023), who claim that HS are successful in encoding/decoding relationships and constructing pragmatically appropriate utterances. The authors also report that HS are not always the mirror images of the MS group, yet none of the differences turn out to be significant, indicating only numerical differences. This might be related to the design of the experiment. Yılmaz and Sauermann (2023) asked their participants to do an elicitation task where they were asked first to read a dialogue and then to choose their preferred form of the NP (accusative-marked indefinite NP, unmarked indefinite NP, and accusative-marked definite NP) with no time limitation. This indicates that the dialogue and all the possible choices were visible to the participants, which provides them the opportunity to read the dialogue again before deciding on the correct answer. On the other hand, the present study employs a word-by-word noncumulative moving window paradigm. In this design, the participants read the sentences by pressing a button that masks the previous word and makes the next word visible. Therefore, HS had to read/process the two sentences presented to them in a word-by-word fashion, and when they reached the end-of-sentence question, they could only see the question on the screen but not the sentences that they had read/processed. This means that HS had to remember the discourse/pragmatics cue presented in the context sentence, remember the definiteness manipulation of the NP in the continuation sentence, and then use their metalinguistic knowledge to make a judgment about the sentences they read. All these cognitive demands might be the reason why the present results differ from the results of Yılmaz and Sauermann (2023).

The HS group's performance in the end-of-sentence response data for context sentences with plural NPs reveals a restructuring and simplification in their grammars (Putnam and Sánchez, 2013) as a result of the “accusative-marked indefinites” being a late-acquired and insufficiently activated grammatical feature. Additional support for the restructuring and simplification process in the heritage grammar comes from Putnam (2019), who argued that HS develop unstable and unconsolidated grammars, resulting in divergent performances when compared to MS. A recent proposal by Polinsky and Scontras (2020) provides further evidence for these claims. According to this proposal, HS have limited processing resources, and this limitation leads them to restructure their grammar in a way that frees up processing resources. The limited nature of their processing resources, together with the added cost of operations in their non-dominant language, forces HS to restructure the grammar of their heritage language in a less ambiguous, more regular, and less structured way. For the present study, “the restructuring of grammar” means that HS try to regularize the definiteness system by not considering the plurality of the NP in the context sentence. When all these proposals are taken into consideration, the restructuring/simplification of heritage grammar might explain why the HS group displays vulnerability/difficulty to the pragmatically appropriate choice only in the end-of-sentence response data and only for context sentences with plural NPs. This means that HS have vulnerability/difficulty in integrating morphosyntactic and discourse/pragmatic knowledge in the end-of-sentence acceptance percentages only in plural NP contexts. The finding that HS experience vulnerability/difficulty when they have to integrate external interfaces is not novel and supports the claims of the IH, which in the present study is observed only for indefinite-specifics in plural NP contexts.

All in all, while HS do not differ from the MS group in reading/processing the experimental sentences, the end-of-sentence response data indicates significant differences between the groups only in indefinite-specific sentences following a context sentence with a plural NP. The MS group shows sensitivity to plural NP contexts and choses pragmatically appropriate indefinite-specific sentences significantly more, whereas the HS group does not display this sensitivity. This difference between the HS and MS groups can be explained by the late acquisition and lack of activation of indefinite-specific structures together with the restructuring/simplification of grammar (Putnam and Sánchez, 2013; Putnam, 2019; Polinsky and Scontras, 2020) that HS employ to overcome the vulnerability/difficulty they experience when they have to compute the external interfaces so that they can make the correct judgment regarding the sentences they read. Although HS seem to differ from MS in offline tasks, as revealed in this study and as has been attested repeatedly in previous literature, the online measure, which is the novelty of the present study, shows that they do not differ from the MS group in real-time processing. This is essentially what this study contributes to the literature. However, to be able to reach more generalizable results about the HS group's online processing patterns, more online processing experiments on external interfaces need to be carried out to unveil how HS process their heritage language in real time and compare their performance to the MS group to see if HS really have processing resource limitations and, if so, how these limitations affect their language processing. Considering that end-of-sentence question responses may offer further information on sentence processing, it appears more beneficial to include these questions after each experimental item. Only then can we obtain more in-depth knowledge, which will enable us to draw more generalizable conclusions.

## 5 Conclusion

The present study focuses on Turkish definiteness, which involves the integration of a linguistic and a non-linguistic domain, also labeled as external interfaces, and investigates if the integration of these two domains is vulnerable/difficult for HS in comparison to the MS group by employing a self-paced reading experiment. The results demonstrate a parallel performance in the online reading time data but only partial differences between the two groups in integrating the external interfaces in the offline end-of-sentence response data. Future research on HS should focus on different phenomena that involve not only external but also internal interfaces and test these phenomena via online experimental methods to see how HS process their heritage language under time pressure. This will enable the researchers to understand the nature of HS and their heritage languages and to get a complete picture of theories regarding heritage languages and their speakers.

## Data availability statement

The raw data supporting the conclusions of this article will be made available by the authors, without undue reservation.

## Ethics statement

The studies involving humans were approved by University of Potsdam Ethics Committee. The studies were conducted in accordance with the local legislation and institutional requirements. The participants provided their written informed consent to participate in this study.

## Author contributions

SU: Writing—original draft, Writing—review & editing, Resource, Formal analysis.

## Footnotes

^1^When the word ends with a consonant, *-I* and its vowel harmony variant (*-i, -**ı, -u*, and *-ü*) are added, and when the word ends with a vowel, *-(y)I* and its vowel harmony variant (*-(y)i, -(y)**ı, -(y)u*, and *-(y)ü*) are added.

^2^Abbreviations used for morphemic glosses: 1, first person; 3, third person; ABL, ablative; ACC, accusative; DAT, dative; GEN, genitive; LOC, locative; PL, plural; POSS, possessive; PST, past tense; REL, relative clause; SG, singular.
